# Prevalence and Barriers to Ending Female Genital Cutting: The Case of Afar and Amhara Regions of Ethiopia

**DOI:** 10.3390/ijerph17217960

**Published:** 2020-10-29

**Authors:** Sintayehu Abebe, Muluken Dessalegn, Yeshitila Hailu, Misrak Makonnen

**Affiliations:** 1 Amref Health Africa in Ethiopia, Reproductive Maternal Child and Adolescent Health Department, Addis Ababa P.O. Box 20855 Code 1000, Ethiopia; 2 Amref Health Africa in Ethiopia, Monitoring, Evaluation and Research Department, Addis Ababa P.O. Box 20855 Code 1000, Ethiopia; mulusef@yahoo.com; 3 School of Nursing and Midwifery, Western Sydney University, Blacktown, NSW 2751, Australia; 4 Amref Health Africa in Ethiopia, Addis Ababa P.O. Box 20855 Code 1000, Ethiopia; YESHITILA.HAILU@Amref.org; 5 Amref Health Africa in Ethiopia, Addis Ababa P.O. Box 20855 Code 1000, Ethiopia; Misrak.Makonnen@Amref.org

**Keywords:** female genital cutting barriers, socio-ecological model, cross-administrative border

## Abstract

Female genital cutting (FGC) remains highly prevalent in Ethiopia, in spite of a slowly decreasing trend over the last decade. In an effort to inform and strengthen FGC interventions in Ethiopia, this study aimed to assess FGC prevalence in cross-administrative border* districts and to explore barriers to ending FGC. A mixed methods, cross-sectional study was employed in three districts in the Afar and Amhara regions in Ethiopia. A sample of 408 women with female children under the age of 15 were included in the study. Additionally, 21 key informant interviews and three focus group discussions were held with local government officials and community stakeholders. The study found that the prevalence of FGC among mothers interviewed was 98%. Seventy-four percent of the female children of participants had undergone FGC. Of the youngest (last born) female children, 64.7% had experienced FGC. The participation of respondents in cross-administrative FGC practices ranged from 4% to 17%. Quantitative analysis found that knowledge and attitude towards FGC, level of literacy, place of residence, and religious denomination were associated with FGC practice. The study also found that the lack of participatory involvement of local women in programs that aim to end FGC and the lack of suitable legal penalties for those who practice FGC exacerbate the problem. A significant proportion of participants support the continuation of FGC practices in their communities. This finding indicates that FGC practice is likely to persist unless new approaches to intervention are implemented. It is recommended that a comprehensive response that couples community empowerment with strong enforcement of legislation is administered in order to effectively end FGC in Ethiopia by 2025, in alignment with the national plan against Harmful Traditional Practices. * Cross-administrative border means a border between two regional states in Ethiopia. In this document, it refers to the movement of people between Amhara and Afar regional states.

## 1. Introduction

The World Health Organization (WHO) defines female genital cutting (FGC) as all procedures involving partial or total removal of the external female genitalia, or other injury to female genitalia for non-medical reasons. FGC is widely recognized as a violation of human rights, including the rights to health, security, and physical integrity, and the right to be free from torture, cruel, inhumane, or degrading treatment. The right to life is also compromised given that FGC commonly results in increased morbidity and mortality of girls and women across the world. The practice of FGC is a reflection of deep-rooted gender inequality and constitutes an extreme form of discrimination against girls and women [1,2].

Globally, more than 200 million girls and women alive today have undergone FGC across 30 countries in Africa, the Middle East, and Asia where FGC practice is concentrated [1]. Three million girls are estimated to be at risk of undergoing FGC every year. Although, FGC has been reported in all parts of the world, Sub-Saharan Africa is disproportionally affected by high prevalence. Some of the highest prevalence rates reported are in East Africa, including Somalia (98%), Djibouti (93%), and Mali (89%). It is estimated that there are over 83 million women who have experienced FGC living in Sudan, Nigeria, Egypt, and Ethiopia alone [3].

According to the 2016 Ethiopian Demographic Health Survey (EDHS), 65% of women in Ethiopia between the ages of 15–49 years old have undergone FGC [4]. The prevalence is highest among the ethnic groups of the Afar (98%) and Somali regions (99%), followed by the Wolaita and Hadiya (92% prevalence for both) [4]. The EDHS reports FGC is more prevalent among women with lower socioeconomic status. Women living in rural populations had a higher prevalence of FGC (68%) compared to urban populations (54%), and women with lower education attainment and wealth also experienced more FGC [4]. Findings from the EDHS also suggest young girls between the ages of 0 to 15 years old are more commonly subjected to FGC. Nearly half (49%) of the women that underwent FGC were cut when they were below the age of five. Twenty-two percent of EDHS respondents reported being cut between the age of five and nine years old, 18% between the age of 10 and 14 years old, and 6% at the age of 15 or older [4]. The age at which FGC is performed varied among respondents due to local traditions and other circumstances [5].

Despite international and national efforts to eliminate FGC, the prevalence of FGC in Ethiopia remains high [1,5,6,7,8,9,10,11,12]. Over the last two decades in particular, Ethiopia has taken significant legal, policy, and programmatic measures to eliminate FGC. The country has also developed a national Harmful Traditional Practices (HTPs) strategy that aims to take a three-pillar approach (prevention, provision, and protection) to fulfilling its commitment to ending FGC and early/child marriage practices by 2025 [6,7,8,9,10].

Ethiopia has witnessed an overall decline in FGC over the last decade, however the practice remains rampant in many parts of the country [4]. Research on FGC in Ethiopia is limited, with most data available limited to survey data reported by the EDHS every five years. Additionally, the major barriers that deter progress towards ending FGC are not well documented. To expand the literature on FGC in Ethiopia and to strengthen intervention efforts on the ground, this study aimed to assess district-level prevalence of FGC, the influence of cross-administrative borders, and barriers to ending FGC in the Afar and Amhara regions of Ethiopia.

## 2. Materials and Methods

### 2.1. Study Design

A cross-sectional analysis using mixed methods for data collection was conducted. Quantitative findings from survey data were substantiated and triangulated using qualitative data collected through focus group discussions (FGD), key informant interviews (KII), and fishbone analysis. A thorough desk review of available international conventions and treaties, policy, legal frameworks, and implementation guidelines related to FGC in Ethiopia were also reviewed and assessed in part of the study.

### 2.2. Conceptual Framework

**Figure 1:**
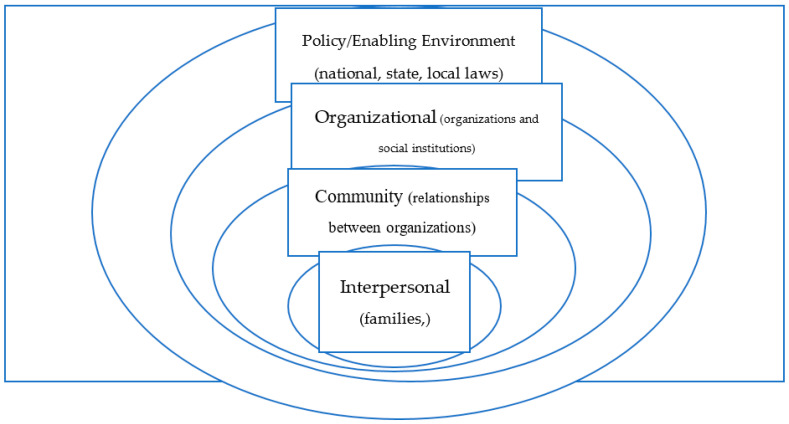
Socio-Ecological Model, (Source: CDC Official website) that affects to end FGC.

The study was designed using a critical gender lens, framed by the Socio-Ecological Model (SEM) (Figure 1). SEM is a theory-based framework that considers the multifaceted and interactive effects of personal and environmental factors on behavior. There are four nested, hierarchical levels of the SEM: interpersonal, community, organizational, and policy/enabling environment [11,12] (Figure 1). The model makes clear that effective intervention acts simultaneously across all levels. In the context of FGC, the practice is heavily influenced by societal factors, such as gender inequity, and sustained by intersecting drivers at the interpersonal and community levels, such as stigma and cultural norms for example.

### 2.3. Study Setting

The study was conducted in three adjacent districts (Kewat, Telalak, and Dewe) in the Amhara and Afar Regional States. Kewet district is located in the North Shoa Zone of the Amhara Region and has a population of 95,895. Telalak has a population of 46,607, and Dewe a population of 51,964, both located in the Afar region [13]. The two regions were purposively selected to introduce an intervention on FGC and this study helps to give an input for potential intervention. The geographical proximity of each of the districts offers an opportunity for cross-regional learning, especially given the pastoralist lifestyle of the Afar in which they frequently migrate (temporarily) to the Amhara region in search of pasture and water for their livestock. Even though the Amhara and Afar populations differ in culture and religion, they share socio-economic similarities and are structurally and socially interlinked.

### 2.4. Study Population

Mothers (15 to 49 years old) living in the three districts who have at least one female child under the age of 15 years old were the primary targets for participation in the study. FGD and KII, on the other hand, included men over the age of 18 years old, and representatives from District Health Offices in the targeted study areas. Key informants included law enforcement bodies, anti-HTP committee members, religious leaders, female genital cutters, and local-level officials from the women and children’s bureau.

### 2.5. Sample Size and Sampling Procedure

Quantitative sample size: The total sample size calculated was 408 participants. The sample size was calculated using Epi-info version 7.0.9.7 software by considering the single population proportion formula based on the following assumptions: proportion of under 14 girls undergoing FGC in Afar region 15.7% (EDHS 2016), 95% level of confidence, 5% precision, and 15% non-response rate. Regional figures reported by the 2016 EDHS were used as a reference to determine the maximum sample size.

Qualitative sample size: The sample size for the qualitative approach to the study was determined using the scientific principle of the state of saturation. The researchers collected the data until similar information was coming out from study participants. The state of saturation after twenty-one key informants (seven KII per district), three FGDs, and two participatory assessments were conducted.

Sampling procedure: With a sample proportion of 15%, two kebeles from each district were included in the study. One was selected in consideration of the research aim which sought to assess the influence of a cross-administrative border on FGC, and the second was selected using the lottery method. A sample of 408 was divided between each of the Kebeles based on probability proportional to size (PPS). Households with children under the age of 15 years old were used as a sampling frame for the study. A systematic method of random selection was then used to reach the final study units.

### 2.6. Data Collection Tools and Methods

Data colllection tools were developed and prested before data collection for both qunative and qualitative assessemnt. Below you see the details process conducted for data collection.

Document/desk review: A desk review template was used to review all relevant documents, including, but not limited to, policy, legal frameworks, reports, and government implementation guidelines.

Community-level household survey: Mothers/caretakers were interviewed regarding their youngest female child to experience FGC (index case). If more than one child had experienced FGC, only the youngest case was selected to avoid duplication of information and to reduce recall bias. Household (HH) data was collected electronically through ODK/KOBO using a structured questionnaire with pre-coded answers.

Key informant interviews (KII): A semi-structured interview guide was used to facilitate in-depth, key informant interviews. A total of 21 key informant interviews were conducted with representatives from legal offices, health bureaus, the women and children’s bureau, the education bureau, health workers, and religious leaders. All interviews were tape-recorded with the consent of each participant.

Focus group discussions (FGDs): Three FGDs were conducted in the targeted communities consisting of eight participants (women caretakers) in each group.

Participatory assessment of development (PaDEV): Two PaDEVs (one from each region) were conducted to identify the root causes of FGC and to fill in the Fishbone analysis with relevant key partners and government offices.

### 2.7. Data Quality and Assurance Mechanisms

The research team took extensive measures at various stages of the study (data collection, data entry, and analysis) to ensure data quality. Before the commencement of data collection, the study team took into consideration the use of highly consulted instruments and the recruitment of experienced data collectors and supervisors with the ability to speak the local languages of the study areas. All data collectors were trained on the tools to foster a common understanding and reduce bias. The questionnaire uploaded to kobo-toolbox made each question ‘required’ to avoid variation and unanswered questions. Pretested and amendment was made ahead of the full data collection. During the data collection stage, filled questionnaires were checked for completeness and consistency on-site and using an electronic data collection device. Text editing and rigorous data cleanup was conducted after all data were collected.

### 2.8. Quantitative Data Management and Analysis

Each completed survey was managed centrally; the quantitative data from the kobo toolbox was exported to SPSS version 25 software for analysis. The findings were presented using text, descriptive statistics, frequencies, tables, and graphs. Multiple logistic regression was used to explore barriers for FGC. The analysis was done using SPSS.

### 2.9. Qualitative Data Analysis:

The qualitative data were encoded to AtlasTi software and the analysis followed a thematic framework. It involved thematic coding of transcribed and translated in-depth KII and FGDs. A hybrid coding approach, which includes the process of creating pre-set and emergent codes, was used. Ideas, concepts, actions, relationships, and meanings that evolved from the data that were different from the pre-set codes were used as the emergent codes. Data was then analysed using a thematic approach by conducting an ongoing content analysis. In general, the qualitative data analysis followed the five interrelated steps: reading, coding, displaying, reducing, and interpreting.

### 2.10. Triangulation

In order to ensure the reliability and credibility of the data, different methods and sources of data were used to triangulate findings. Triangulation allows for the enhancement of rigour, breadth, richness, and depth of the inquiry, and also reduces the potential for bias.

### 2.11. Ethical Considerations

Ethical approval for this operational research was obtained from Semera University of the Afar Region following a letter of permission from the Afar and Amhara Regional Women, Children and Youth Bureaus. Study participants were assigned unique ID numbers to avoid the use of names and any other identifiers on any of the principal data collection instruments. All study materials were kept confidential and secure, with access granted only to members of the research team for data management and analysis. Verbal informed consent was obtained from each participant prior to conducting interviews. Individual interviews were conducted in private locations and group interviews were conducted in a way which facilitated free expression of opinion by all participants.

## 3. Results

The first section of the results presents the key findings from the desk review. This review focused on legal and policy frameworks related to FGC, organized using a deductive reasoning approach which starts with analysis of the global context, then narrows in on the local (Ethiopian) context. The second section presents the findings of the survey administered by the research team on district-level FGC prevalence and the influence of cross administrative travel, which was supplemented by qualitative data collection to infer key barriers to ending FGC in Ethiopia.

### 3.1. Key Findings of the Desk Review on Legal and Policy Frameworks, and Strategy Implementation

#### 3.1.1. Global and Regional Context

Following the adoption of the resolution to end FGC by the UN Commission on Human Rights in 1952, many global conventions and treaties have been signed by heads of states to stop FGC. The World Health Organization (WHO) has issued a joint statement against the practice of FGC together with the United Nations Children’s Fund (UNICEF) and the United Nations Population Fund (UNFPA) in 1997 and efforts have since been made to counteract the practice through changes in public policy, research, and interventions within communities. The global conventions and treaties that condemn FGC, signed by many nations including Ethiopia, are summarized in Supplementary Materials.

In alignment with their global commitments, many countries have revised their legal and policy frameworks and increased political support to end FGC, including a law against FGC in 26 countries in Africa and the Middle East, as well as in 33 other countries with migrant populations from FGC practicing countries. Ethiopia is a signatory of the above frameworks and many other international laws and regulations against harmful traditional practices, in an effort to protect women and children.

#### 3.1.2. The Constitution of the Federal Democratic Republic of Ethiopia

The constitution of the Federal Democratic Republic of Ethiopia (EFDRE, 1995) includes a clause that discourages harmful traditional practices (HTPs) and guarantees women“… the right to protection by the state from harmful customs. Laws and practices that oppress women or cause bodily or mental harm to them are prohibited.” The constitution prohibits violence against women and girls stating that a person has the right to be protected from bodily harm. However, it does not explicitly prohibit the act of female genital cutting (FGC).

#### 3.1.3. National Legislations

The Government of Ethiopia has banned HTPs, particularly FGC, and has proclaimed legislation for its enforcement. The criminal code of Ethiopia underscores that despite respect for the culture of its people, it has put in place legal provisions for HTPs, including FGC, with penalties for offenders. The revised Penal Code (EFDRE, 2005) addresses FGC, domestic violence, abduction, early marriage, and trafficking. The revised code, Articles 569 and 570 of the law state “a parent or any other person who participates in the commissioning of FGC practice, organizing or taking part in any movement that promotes FGC or encourages someone to disregard the legislation prohibiting harmful traditional practices including FGC is punishable with simple imprisonment not exceeding three months, or payment of a fine not exceeding 500 Birr…. Anyone who sews up a woman’s vagina will face 3–5 years of imprisonment, and if a health hazard is caused, he or she will face 5–10 years of severe imprisonment” (EFDRE, 2005).

As stated in Article 569 and 570, Ethiopian criminal law makes clear practicing FGC on a woman of any age is punishable by law. However, FGC is not explicitly called out in other relevant articles of the Ethiopian criminal code. This inconsistency leaves room for discretion in legal proceedings, which can make women even more vulnerable to the harms of FGC. For example, it can be inferred that cross-border FGC in Ethiopia is illegal, even though there is no explicit discussion of cross-border FGC in Ethiopian law. Article 18 of the penal code applies to any person who commits a crime outside of Ethiopia against an Ethiopian national, and to any Ethiopian national who commits a crime under the Code outside of Ethiopia, with provision for extradition. Hence, if FGC is performed on an Ethiopian in another country or to a woman from another country by an Ethiopian, it is punishable under Ethiopian law. Similarly, the law also penalizes failure to report FGC, despite not explicitly mentioning FGC in the Article. Article 443(1) states that “anyone knowing the commission of, or the identity of the perpetrator, a crime punishable with death or rigorous imprisonment, fails to report such things to the competent authorities is punishable with a fine not exceeding 1000 Birr, or by simple imprisonment not exceeding six months.” Ethiopian law also fails to protect uncut women (and their families) who commonly face severe verbal abuse and/or exclusion from society. Similarly, it fails to explicitly criminalize the medicalization of FGC and any health professional found performing or reported to have performed FGC. However, in January of 2017, The Ministry of Health (MoH) recognized FGC as a violation of human rights and took a step forward by banning the medicalization of FGC in all public and private medical centres and facilities (21).

#### 3.1.4. Strategy and Implementation Documents

Over the last three decades, Ethiopia has developed several policies and strategies, and has taken institutional measures to ending FGC in Ethiopia. The National Policy on Ethiopian Women (1993) and National Strategy and Action Plan on HTPs against Women and Children in Ethiopia (2013) employ three strategic pillars, namely prevention, protection, and provision, with specific interventions to end child marriage and FGC under each of the pillars. The National Costed Roadmap to End Child Marriage and FGC 2020–2024 is a key guiding document. It outlines the need for awareness-raising on legal frameworks, the involvement of local and religious leaders, and the involvement of key interpersonal stakeholders such as mothers and traditional practitioners. The Roadmap also has detailed monitoring, evaluation, and accountability guidelines with a clear strategy on reporting and communication. It uses a multi-sectorial collaboration approach with its implementation being coordinated by the Ministry of Women, Children and Youth, with its partners.

The National Social Protection Strategy of Ethiopia 2016 also calls for robust communication and awareness raising plans for the prevention of abuse, violence, neglect, and exploitation of women, including FGC.

### 3.2. Prevalence, Cross-Administrative Border Experience and Barriers to Ending FGC

#### Socio-Demographic Characteristics of Study Participants

A total of 405 respondents participated in the study, indicating a 99.2% response rate. The sample was distributed to 201 women in Amhara and 207 women in the Afar region. More than three quarters (79%) of the respondents reside in rural areas and over half (59%) of the respondents identify as Muslim, and 41% as Orthodox Christian. The majority (92%) of respondents were married and worked in the home as homemakers (67%). The mean age of the respondents was 31 years old and the average number of female children was 2.41 per woman (Table 1).

### 3.3. Prevalence of FGC

**Figure 2:**
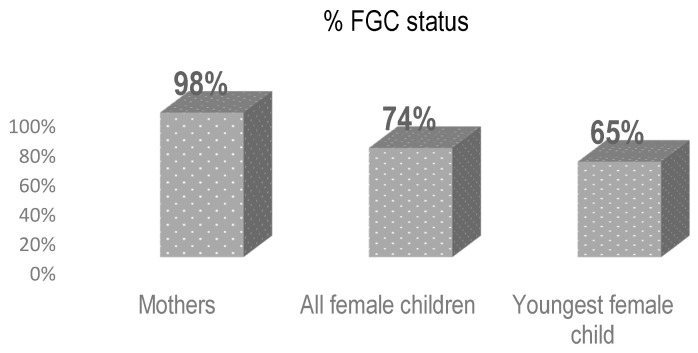
FGC among Female family members, Afar and Amhara April 2020.

The study found that almost all women participating in the survey 398 (98%) underwent FGC (Table 2). The prevalence of FGC among all female children of the participants was 796 (74%). Sixty-five percent of the participants youngest daughters (last born) have undergone FGC (Figure 2). Most (72%) were cut under the age of one. Although the trend in prevalence shows a slight decrease from figures reported by mothers, the prevalence of FGC in these regions remains high. 

At the household level, the study found that about 26.9% of mothers did not have FGC performed on their children. Of the 74% who did undergo FGC, most were cut by a traditional cutter (72%) followed by 8.9% who were cut by a traditional birth attendant (Table 2).

### 3.4. Education and Occupation of Study Participants

The study found that the education attainment of participants was associated with decreased FGC. In both Afar and Amhara Regions, the youngest child’s FGC status decreased as the educational status of their mother increased. In other words, prevalence of FGC among the youngest child was found to be high (79.8%) in mothers with no education.

More than two-thirds (71%) of women participants self-identified as illiterate in addition to 80% of their partners. Similarly, 26% of women respondents had completed a primary education, meanwhile 18% of their partners had a primary level education. Both women and their partners who had a secondary level education were less than 3% of the total respondents. As depicted in Table 3, the vast majority of women worked in the home as homemakers (66%) followed by just over one-quarter of respondents working as farmers (28%). The study also found that rural residency was an independent predictor for the practice of FGC. Consequently, mothers who live in rural areas were 3.3 (AOR = 3.33, 95%CI: 1.81–6.12) times more likely to practice FGC than urban residents. Mothers residing in rural areas were also seven times more likely to support FGC continuation in the future (AOR: 7.12, 95%CI: 1.91–26.52).

Findings from the qualitative study indicate that the practice has declined over time and changed from the most severe type of FGC (which includes suturing the external genitalia) to a less severe type of FGC due to increased knowledge of FGC-induced complications such as bleeding, pain during intercourse, and painful delivery. Most key informants and focus group participants, especially the perpetrators of FGC and religious leaders, confirmed witnessing the death of many children during FGC, adolescent girls’ experiencing severe pain during menses, labour complications which requiring referral to higher health facilities, and aggravated maternal death. The enforcement of the revised penal law on FGC has contributed to the slight reduction in FGC practice. At the same time, mothers in the study stated that Traditional Birth Attendants (TBAs) practice FGC less frequently because they have been instructed to stop doing so. As a result, many have resorted to practicing FGC at home either by the mother herself or by the grandmother. “What is happening these days is: people practice FGC hidden inside their home without calling anyone. Previously, many people were invited to attend during the procedure, but now only the mother of the victim and the cutter are there. which makes the identification and reporting very complicated” (Religious leader: Key Informant, Afar)

### 3.5. Cross-Administrative FGC Experience

In exploring cross-administrative FGC experiences, the study found that women occasionally travel from their homes to other locations to look for FGC performers for their female children. Of the 64.7% of participants who had their youngest child undergo FGC, 96% of the girls were cut in their residence, whereas about 4% reported taking their child to another location for the practice.

About 17.3% of the study participants stated that members of other communities bring children to their village to practice FGC (from Afar to Amhara or vice versa) and about 5% confirmed that members from their own community take their children to other areas for FGC. The main reason for cross-administrative border FGC practice was that respondents perceived the law to be stricter in their home village/area (93%; 14) (Table 4).

Travelling to another districts/region for FGC was relatively more significant in the Afar region (6.3%) compared to the Amhara region (0.5%). It is highly likely that the prevalence of cross-administrative border FGC is underreported. Participants showed hesitancy when discussing cross-administrative border FGC and were more responsive when asked about other people as compared to questions about themselves.

A FGD participant suggested that if a cutter refuses to perform FGC, community members simply bring a cutter from another area to their home: “FGC is being practiced after seven days of delivery. it is customary that the mother and neonate do not come out of their house/room for six weeks after delivery. but if there were no cutters or there was a refusal from a cutter, we would bring another cutter from another area” (mother, FGD Discussant, Afar).

The move away from a more severe form of FGC (excision and infibulation) has been promising, however the link between religious beliefs and the clitoridectomy or “sunna” form of FGC has been a significant barrier to ending FGC. There is a widespread belief that this form of FGC is a religious requirement: “We are currently practicing the ‘sunna’ or cutting only the clitoris and we can’t stop doing it because our religion orders us to do it “(mother, FGD discussant in Afar). Religious sensitivity has also presented challenges to the enforcement of legal measures against those who engage in FGC.

In Kewot district of the Amhara region, FGC practice has not been as widespread as it has been in the past. A traditional birth attendant shared: “as to my knowledge, FGC is not being practiced in our community. In the old days, FGC was believed to augment the beauty of girls. However, currently, it is considered a harmful traditional practice. We learned that avoiding FGC makes labour easier and reduces birth complications (50-year-old TBA woman).

### 3.6. Attitude Towards Continuation of FGC

According to sociological theory, childhood experiences influence the trajectory of our adult lives. There is also a growing body of literature on adverse childhood experiences (ACEs) and its impacts on long-term health outcomes. Accordingly, experiencing FGC during childhood can have long-term psychosocial impacts on women, in addition to the physiological outcomes such as painful menses and risk of complications during labour and delivery. In spite of the negative health risks associated with FGC, there remains a widespread positive attitude regarding the continuation of FGC in both the Afar and Amhara regions. Nearly half (48%) of the respondents support the continuation of FGC practices and 47% expressed that they want their future children to undergo FGC. The primary reason for continuing FGC is that it is deeply embedded in the traditions, culture, and values of the communities included in the study. In order for future FGC interventions to result in behavior change, it is critical that the community is equitably included in design and implementation, and that there is a concerted effort to raise factual awareness and influence perceptions and beliefs at the community level.

### 3.7. Barriers to Ending FGC

The study found several barriers ranging from the individual to societal level which act as barriers to the elimination of FGC. These findings are summarized using the socio-ecological model approach below:

I. Individual/family level barriers to ending FGC

A large majority of the women participants have low literacy and low education attainment which limits their knowledge and attitude about FGC and the severity of its impacts. The women generally come from conservative backgrounds in which they hold religious beliefs in support of FGC. FGC has become a cultural practice passed on between generations and one which is deeply ingrained in local customs. There is often internalized stigma at the individual level and restrictive social norms at the family level which enable FGC practice to continue: “in Afar, almost every family practices FGC. If they have a female newborn, they hide what they are doing following a strong belief that she will not get married in the future, if not circumcised, and will get stigmatized in the community. In Afar, ‘Sunna’ is considered as part of Islamic religious obligation and worth practicing. Some families force TBAs to practice the ‘Sunna’ type of FGC.” (Mother of a circumcised child, FGD discussant, Afar).

The interlinkage of FGC with cultural and religious beliefs poses a significant obstacle to ending FGC and to implementing legal repercussions. As a result, FGC intervention has had less emphasis on law enforcement, and more so on health education and awareness creation across disciplines and stakeholders. These activities are often unsuccessful because they are not designed to address behavioral change at the family and community level. Families and community members frequently feel unsafe disclosing the identity of cutters and other families who practice FGC due to stigma and social isolation. Many community members also fear that challenging FGC implies that they are abandoning their culture and tradition. A key informant spoke to the importance of social networks and rapport in their community: “Social relations are very important in our community; cutters and those who circumcise their daughters are living amongst us. Exposing them to face legal punishment such as imprisonment is not good, also you will risk losing your neighbors forever“ (Religious leader: Key informant (Amhara)).

A finding that is often neglected is the influence of mothers on sustaining FGC in their communities. Many mothers carry a more conservative view of the need for FGC in comparison to fathers. This is possibly because women understand firsthand the brunt of the stigma and shame placed on uncut women. This stigma even causes marriages to end in the communities studied. There is an assumption in Afar and Amhara communities that if a woman did not undergo FGC, she will not be considered a virgin and her husband may leave (divorce) her following the consummation of their marriage. An FGD participant expressed fear of what her daughter may face at the time of marriage if she is not cut: “We know that no one will marry our daughter if we didn’t circumcise her. The morning after her wedding, her husband will leave/divorce her saying that she was not virgin. I do not want this to happen to my daughters” (Mother with a circumcised daughter- FGD discussant).

There is also a common belief that an uncut girl may bring bad spirits to the family and the community. Therefore, there is a preference among men and families to marry a woman who has undergone FGC.

II. Community level barriers to ending FGC

Community attitude and stigma: Community members in Afar and Amhara predominately have conservative views of sexuality. There is a common belief that FGC decreases sexual desire and prevents women from engaging in extramarital sex. A police officer interviewed in Afar described some women feeling shame about their FGC status, which sometimes leads to disruption in their schooling because they refuse to leave the home: “there is a community held belief that if a girl is uncircumcised, she will be overactive in her sexual desire, which is unacceptable by the community. Then, if the community knew that the girl was not circumcised, she together with her family will face stigma where community members say that she is ugly, making the girl feel shame and even refusing to leave her home including to go to school” (Police Officer, Afar).

Misconceptions and factually incorrect views of FGC often go unaddressed in these regions. The pastoralist environment in Afar and the mobile nature of the population makes it difficult to conduct regular health education and awareness creation activities in a sustainable way. In many areas, cutters use FGC as a primary source of income. Challenging FGC and reporting cases to local authorities is viewed negatively as it threatens the livelihood of the cutter and her family. These interconnected factors play a role in decreasing community support for the case reporting process which is critical to law enforcement and legal protection measures.

It is clear that FGC is deeply embedded in the traditions and cultural norms of people around the world, including in Ethiopia. Designing an intervention which challenges longstanding traditions of a community risks resistance and barriers to adoption. To achieve an optimal impact in ending harmful practices with cultural humility, sensitive issues such as the practice of FGC should be dealt with deep consultation and involvement of the target community itself.

Religion: About 54% of the respondents believe that FGC is obligatory to the practice of their religion. In some districts, women consider ‘end FGC’ initiatives government goals which conflict with their religious beliefs and practice. Multivariate logistic regression analysis by the study team found that mothers who believe FGC is a religious order were 24 times more likely to support the continuation of FGC (AOR: 24.66, 95%, (*p* < 0.05), although the confidence interval was wide (CI: 10.71–56.76).

The stance of religious leaders on FGC in inconsistent and presents many discrepancies which confuse community members on the ruling of their religion on FGC. “Some religious leaders say removing part of the clitoris is a ‘Sunna’ practice and others agree as it is not a ‘Sunna’. Here, I can’t say or decide which one is correct. This needs a discussion with both religious leaders supporting opposing ideas and they should reach and disclose a consensus/FETWA” (Kebele leader, Afar) (FETWA means an interpretation based on Islamic rule/interpretation from an Islamic perspective). In these cases, the research team found that individuals tend to support whichever religious leader best aligns with their personal interests regarding FGC.

### 3.8. FGC Increases Marriage Opportunity

More than half of the study participants (55.1%) believe that FGC is a precondition for marriage. Mothers who think FGC is a requirement for marriage were 14 times more likely to support the continuation of FGC in comparison to their counterparts (AOR: 14.61, 95% CI: 6.27–34.03; *p* < 0.05).

Generally, the community believes that women who do not undergo FGC are more likely to engage in extramarital sex. A possible pregnancy outside of marriage is considered shameful and unlawful from a cultural and religious point of view. Oftentimes, there are also differences in the dowry paid to a bride based on FGC status. Women who did not undergo FGC are looked down upon and considered less desirable for marriage: “if a girl exhibits behaviour that is deemed out of the ordinary, then the community will say that it is because she was not circumcised. Besides, I heard the youth talking that they are not going to marry an uncircumcised girl.” (District MCH officer).

There is also a level of shame projected onto parents if they do not arrange for their daughters to undergo FGC. Oftentimes they are labelled “careless” and indifferent to the social and economic trajectory of their daughters which is traditionally linked to marriage. Most families expressed that they practice FGC simply because they believe it is a part of their culture.

Members of the community suggested modeling FGC interventions after education initiatives which used incentives to increase the enrollment of girls in school. Once the government started providing incentives for families who send their daughters to school, parents in the Afar Region started sending their female children to school. “In the past, Afari girls did not attend school and then to change this, the government started to give cooking oil for the family who sent their daughter to school after which all the other families started to send their daughters to school to get the cooking oil. Also, if the same thing will be done for FGC, I mean if there is an award for the uncut girl, I think the same scenario could work to end FGC” (Traditional birth attendant, Afar).

### 3.9. Organizational Level Response

Ending FGC has moved beyond the agenda of just the health sector to a shared goal across multiple sectors at both the national and regional levels. Despite multisectoral commitment, the study found that there is weak integration of actors on the ground which results in ineffective, and even nonoperational, FGC committees.

Many national programs are being implemented at the district level, however they lack the coordination and the human resources required. The workload is more often than not demanding, and it is difficult for a single person to oversee multiple programs. A large portion of the work involves conducting kebele level criminal case evaluation. Although the legal framework for persecuting FGC practice is in place, case reporting requires convincing evidence. “We did criminal case evaluation at kebeles based on the files received from the police, and based on these files, we would prepare training materials on law awareness creation to the community, but I have never seen a single case of FGC reported in the evaluation” (District attorney head, Amhara). FGC frequently goes unreported due to the community level stigma and fears of social isolation previously discussed. Procedurally, the police are expected to report FGC cases, however the same stigma and fears prohibit police from enabling the legal system to work as intended.

Although the current government structure involves 11 sectors, including police, justice, education, health, and environmental protection, they do not share an integrated plan which inhibits a cohesive approach. Overall, findings at the organizational level underscore a significant gap in in the proper utilization of existing structures such as the health extension program and community-level dialogues for increased FGC case identification and reporting: “we can use health development armies or health extension workers to make the identification, as they conduct house to house visits. FGC can be integrated when they go from home to home for post-natal follow-up or other activities after delivery” (District MCH officer, Amhara).

III. Societal level Barriers to end FGC

### 3.10. Societal Perception

In the Afar region, and to some extent in the Amhara region, the local government is not supported in its efforts to report FGC cases locally, and to federal officials. Social norms enable community support to prioritize the protection of culture and customs over government initiatives, including anti-FGC interventions. There are also gaps in integrating the case reporting process with existing informal social structures/institutions. In communities like the Afar Region, it is considered unacceptable to refer a member of the community to a legal body (local government) for wrongdoing. It is preferred that conflict and ‘misconduct’ is addressed informally, within the community using traditional techniques: “in Afar, we will not oust members of our community for imprisonment, even if they did wrong, rather we let them pass through our community level punishment techniques”. (Religious leader, Key informant, Afar)

### 3.11. Knowledge of Key Actors on the Legal Ground of FGC

There was a general consensus among district level representatives from the health, legal, women, children and youth sectors interviewed that national law (and hence regional law) prohibits the practice of FGC. However, most key informants were not aware of the contents of the legal clause. In fact, they had never witnessed a case in which an individual was accused of practicing FGC. “I don’t know any written policy or law which restricts FGC, but we health professionals teach the community considering FGC as a harmful traditional practice which is also forbidden in all religion” (District MCH officer).

Detailed understanding of criminal code and corresponding punishment was limited to representatives from district-level courts: “Yes, there is a policy and following that there is criminal law which limits the practice of FGC. It is clearly defined in the criminal code, Article 5:61, which says anyone who undergoes FGC is punished by not less than three months in jail or more than 500 ETB (15 USD) (article 5: 65)” (District attorney’s head).

### 3.12. Limitations on Legal Procedures

The study suggests that legal protocol, such as the requirement of evidence, can hinder case reporting and law enforcement. FGC often takes place in secret and out of sight. It is unlikely that there are witnesses who can testify in court, further hindering the legal process and making it even more difficult to criminally prosecute FGC.

Some participants expressed feeling that the sentence is not harsh enough to deter cutters from practicing FGC: “I heard yesterday from the court that the penalty ranges from a fine of 500ETB-3000ETB or jail time for about three months. This is not hard for our people, whether it is in terms of money/being in jail” (District women and children affairs officer, Afar). Girls and women who undergo FGC are at risk of severe complications, including infection, excessive bleeding, and even death. In this regard, the level of punishment is not equivalent to the crime (FGC). The research team speculates that the legislative body probably simplified the sentence due to FGC being a longstanding tradition practiced by virtually almost all members of the community. However, findings from KII and FGD suggest that these light legal implications are worth reconsidering.

## 4. Discussion

Despite a decreasing trend, the study found that a significant proportion of young girls (64.7%) in the Amhara and Afar regions are still subjected to FGC. The findings of the study align with the findings of the EDHS 2016 [4], which also witnessed a similar decreasing trend, but found 47% of adolescents still experience FGC.

FGC prevalence was found to be higher in rural areas compared to urban areas. The study found that 20.4% of urban residents underwent FGC, whereas over three-quarters (79.6%) of study participants living in rural areas experienced FGC. Similarly, the EDHS 2016 found an association between FGC and rural residence. More than half (54%) of urban women had undergone FGC, in comparison to 68% of rural women. This finding suggests FGC interventions should strategically target women residing in rural areas.

The slow decline of FGC practice in Ethiopia remains unsatisfactory. FGC practice is deep rooted and intrinsically linked to social norms, culture, tradition, sexuality, gender, health, and human rights. Culture, in particular, plays a vital role in perpetuating its practice.

At the community level, FGC is often justified by beliefs which conflict with the biological basis of sexuality and physiology. In the Afar region, the community supports FGC with non-factual beliefs that FGC deters extramarital sex. This is culturally linked to values of honor and respect, and to beliefs about customary marriage expectations. Parents want to have a ‘respected girl’ who is chaste and perceived by the community as desirable for marriage. Hence, FGC is a HTP which is thought to ‘safeguard cultural belongingness and membership in the local community’ [3]. Another contributing factor to the slow reduction of FGC practice in Ethiopia is a lack of proper linkage between the main actors within the society and factors that directly affect the process of eliminating FGC. Main actors include the local government as a powerful decision-maker, NGOs, informal institutions within communities, family, individuals, and socio-economic institutions. The failure in the coordination and collaboration among these actors could be seen as a failure in action. This finding is consistent with various studies conducted in different countries [3,5].

## 5. Conclusions

FGC, within the broader context of GBV, is a transnational human rights and health concern. A holistic understanding of the complex socio-cultural dynamics of this practice is important for guiding government policy and strategies to better protect women and girls from FGC.

Although there is relatively decreasing FGC prevalence in the study area, there is still a significant proportion of young girls who are subjected to the practice. The findings of the study indicate that FGC is highly likely to persist in the study areas in the future because a significant portion of women support its continuation in their communities. When assessing cross-administrative border FGC, the research team found that community members travel to other areas or regions when FGC laws are perceived as too strict where they live and less rigid in the other region/area. Barriers for ending the practice of FGC are multifaceted and exist at different levels including at the individual, family, and community levels.

It is recommended that a comprehensive response ranging from strengthening the enforcement of legislation against FGC, empowering women through education, raising community awareness, and active involvement of religious leaders and women are implemented as primary measures to reducing FGC practice. These recommendations can only be realized if there is stronger political commitment to supporting the implementation of existing laws, and high-quality training of relevant community and religious leaders takes place. Moreover, cutters are key stakeholders in FGC practices and therefore should be frontline actors in interventions towards ending FGC. Community representatives (trusted by the community) should also be selected to create awareness and to conduct home-to-home visits, including to the homes of mothers who recently delivered.

These actions should be complemented by robust policy frameworks that put women who experienced FGC at the forefront of the fight against FGC. Improved economic integration and participation, increasing access to basic services such as health and education, and addressing discrimination and inequity are key steps towards achieving the goal of ending FGC and protecting women from harmful practices. Given the multidisciplinary nature of the key barriers to ending FGC, it is critical that a multisectoral approach to intervention is developed and adapted by existing interventions.

## Figures and Tables

**Table 1:** Socio-demographic characteristics of the Respondents, Amhara, Afar women, April 2020 (*n* = 405).

Variable		Frequency	Percent
Place of residence	Rural	320	79.0
	Urban	85	21.0
	Total	405	100.0
Region	Afar	204	50.4
	Amhara	201	49.6
	Total	405	100
Religion	Muslim	239	59.0
	Orthodox Christian	166	41.0
	Total	405	100.0
Marital status	Divorced/widowed	28	6.9
	Married	374	92.3
	Single	3	0.7
	Total	405	100.0
Partners Educational status	No education	325	80.2
	Primary education	69	17.0
	Secondary education	11	2.7
	Total	405	100.0
Mothers Occupational status	Farmer	113	27.9
	House wife	266	65.7
	Others	26	6.4
	Total	405	100.0
Ethnicity	Afar	192	47.4
	Amhara	201	49.6
	Oromo	12	3.0
	Total	405	100.0
Mean age of mothers	31.2 ± 7		
Mean number of female children	2.41 ± 1.3		

**Table 2:** The experience of FGC for the mother and the children, Afar and Amhara, April 2020 (*n* = 405).

Characteristics	Response	Frequency	Percent
Mothers FGC status	No	7	1.7
	Yes	398	98.3
	Total	405	100.0
Mothers who have at least one female child cut	Yes	296	73.1
	No	109	26.9
	Total	405	100
Total female children FGC status	Yes	796	74.2
	No	252	25.8
	Total	978	100
	Mean ± SD *	2.41 ± 1.3	
The youngest girl underwent FGC	No	34	8.4
	Yes	262	64.7
	Total	405	100.0
FGC Performed by	Traditional cutters	295	72.8
	Traditional birth attendant	36	8.9
	I don’t know	70	17.3
	Others	4	0.9
	Total	405	100.0

* Standard Deviation (SD).

**Table 3:** Comparison: Education, Age at time of FGC by region, Amhara, Afar women, April 2020 (*n* = 405).

Characteristics	Youngest Child Circumcision Status				Total
Mothers Educational status	Response	Not youngest	Yes	No
	No education	56	209	23	288
	Primary Education	47	47	11	105
	Secondary Education	6	6	0	12
	Total	109	262	34	405
	Age range of Circumcision				Total
Region			<1 year	≥1 year	
	Afar	2	189	15	206
	Amhara	141	0	58	199
Total		143	189	73	405

**Table 4:** The experience of women and child FGC, Afar, Amhara, April 2020 (*n* = 405).

Characteristics	Response	Frequency	Per Cent
Where the FGC practice took place?	Around my residence	283	96%
	Far away from my residence	13	4%
	Total	296	100%
In which region did the last child’s FGC occur?	Afar	196	66%
	Amhara	100	34%
	Total	296	100%
Do you think a member of another community brings their children to your community for FGC?	No	335	82.7
	Yes	70	17.3
	Total	405	100
Does a member of your community take their children to Afar or Amhara for FGC	No	385	95.1
	Yes	20	4.9
	Total	405	100
Did you travel to another district/region for FGC?	No	391	96.5
	Yes	14	3.5
	Total	405	100
What was the reason for crossing your administrative border	The law is stricter in our village	14	93%
	To have girls cut more discretely	1	7%
	Total	15	100%
Do you support the continuation of FGC practice?	No	208	51.4
	Yes	197	48.6
	Total	405	100.0
Would you want your daughter(s) to be circumcised?	No	216	53.3
	Yes	189	46.7
	Total	405	100.0

## Supplementary Materials

The following are available online at https://www.mdpi.com/1660-4601/17/21/7960/s1.
