# Supplementary material for: Prevalence and Barriers to Ending Female Genital Cutting: The Case of Afar and Amhara Regions of Ethiopia
Source: Int J Environ Res Public Health. 2020 Oct 29;17(21):7960. doi: 10.3390/ijerph17217960 (PMC7663154; doi:10.3390/ijerph17217960)
Supplement: Supplementary file 1 [file ijerph-17-07960-s001.pdf]

Supplementary Materials

Supplementary Table 1: Global and Regional FGC conventions and legal frameworks

Conventions and treaties to end FGC	Key points
UN Commission on Human Rights (1952)	Adopted a resolution on ending FGC
Convention for the Elimination of All Forms of Discrimination against Women (CEDAW) (1979)	An important milestone in recognizing FGC's implication on the human rights of victims. The UN General Assembly adopted the Convention on the Elimination of All Forms of Discrimination against Women (CEDAW) in 1979. It clearly defined what constitutes discrimination against women and set up an agenda for national action to end such discrimination. By accepting the Convention, States committed themselves to undertake a series of measures to end discrimination against women.
The UN Convention on the Rights of the Child (CRC) (1989)	The procedure has been identified as both a harmful traditional practice that compromises a child's right to the highest attainable standard of health and a form of violence that is against the best interest of the girl child
The African Charter on the Rights and Welfare of the Child (1990)	Calls for protection against Harmful Social and Cultural Practices including Female Genital Mutilation
African Charter on the Rights and Welfare of the Child (1990)	It calls for states to take all appropriate measures to eliminate 'harmful social and cultural practices affecting the welfare, dignity, normal growth and development of the child'. Besides, the African Charter on the Rights and Welfare of the Child (1990) is built on the African Charter on Human and Peoples' Rights. Child marriage is prohibited, and States agree to

	protect children from trafficking, sexual exploitation and sexual abuse through prostitution or pornographic activities.
The Program of Action of the International Conference on Population and Development (ICPD) (paragraph 4.22) (1994)	According to the ICPD program of action: "Governments are urged to prohibit female genital mutilation wherever it exists and to give vigorous support to efforts among non-governmental and community organization and religious institutions to eliminate the practice"
The Beijing Declaration and Platform for Action (1995)	The Beijing Declaration (1995) was adopted in support of CEDAW and other international human rights treaties. The Platform of Action that was adopted at the conference deals with twelve critical areas of concern: poverty, education, health, violence, armed conflict, the economy, power and decision making, institutional mechanism, human rights, the media, the environment, and the girl child.
A World Fit for Children – UN General Assembly Special session (2002)	Calls for an end to "harmful traditional or customary practices, such as early and forced marriage and FGC
Protocol on the Rights of Women in Africa or the Maputo Protocol (2003)	The Protocol to the African Charter on Human and Peoples' Rights on the Rights of Women in Africa was adopted in Maputo, Mozambique (2003). The protocol stipulates that States shall combat all forms of discrimination against women through appropriate legislative measures. It contains provisions such as elimination of HTPs including FGC and protection of female victims of HTPs. This Protocol also calls for the elimination of all forms of gender-based violence and explicitly prohibits FGC.
Protocol on the Rights of Women in Africa (adopted by AU in 2003)	call for an end to harmful traditional practices

End-FGC-European Campaign (2009)	This campaign contributes to an international mobilization that recognizes the need to join forces to end the practice of FGC
The Inter-Africa Committee on Traditional Practices adopted a Common Agenda for Action (2010)	Contributed to the Zero Tolerance for FGC initiative and declared February 6 as the International Day of Zero Tolerance against FGC

Supplementary Table 2: Ethiopian FGC legal and policy frameworks, 2020

National legal/ policy framework	Provision/protection
National Policy on Ethiopian Women 1993	One of the three objectives in the national policy directly focuses on addressing HTPs, it states, "Eliminating prejudices, customary and other traditional practices that reflect male supremacy. It promotes enabling women to hold public offices and to participate in the decision-making processes at all levels". The following strategies of the policy also support the elimination of HTPs:<br><br>Strategy 6: The government, with cooperation from the people of Ethiopia, shall facilitate conditions conductive to the informing and educating of concerned communities about harmful practices such as circumcision and the marriage of young girls before they reach puberty; and<br><br>Strategy 8: Maximum effort shall be made to eliminate, step by step, prejudices and customary practices based on the idea that women are inferior to men and to repeal all national penal provisions which constitute discrimination against women.
Health Policy of the Transitional Government of Ethiopia 1993	The policy determines that health education and family health services shall identify and discourage HTPs.
Constitution of the Federal Democratic Republic of Ethiopia 1995	Article 16: The right of the security of the person states that everyone has the right to protection against bodily harm, and thereby protects girls and women from VAWG and FGC that constitutes the most severe form of VAWG.
Criminal Code 2005	Article 565: Female Circumcision; Article 566: Infibulations of the Female Genitalia; the punishment of FGC: Article 434. I Article 569. Participation in Harmful Traditional Practices (HTPs).

National Strategy and Action Plan on HTPs against Women and Children in Ethiopia 2013	The Strategy institutionalizes national, regional and grassroots level mechanisms by creating an enabling environment for the prevention and elimination of all forms of HTPs, and to ensure multi-sectoral mechanisms are available to support women and children through prevention, protection and provision/responsive services
Health Sector Transformation Plan 2015/16– 2019/20	The plan recognizes that maternal mortality and morbidity, and health concerns such as fistula are still very high and are consequences of HTPs which it considers as threat to Health.
The Second Growth and Transformation Plan (GTP II) 2015/16–2019/20	GTP II aims to eliminate violence and HTPs including FGC, early marriage and childbearing, GBV, and forced marriage. Among the strategic pillars of GTP II, the eighth pillar aspires to "Promote women and youth empowerment, ensure their participation in the development process and enable them equitably benefit from the outcomes of development".
National Social Protection Strategy of Ethiopia 2016	The strategy targets adolescent girls and promotes communication and awareness raising for the prevention of abuse, violence, neglect and exploitation, including FGC "to maximize impacts on educational outcomes and reduction in child marriage".
Ethiopian Women's Development and Change Strategy and Package 2017	The Strategy addresses HTPs through guaranteeing women's participation and benefits in the social sphere; it calls for activities to prevent HTPs; taking action against perpetrators; provision of the necessary support for victims, giving special emphasis to FGC
